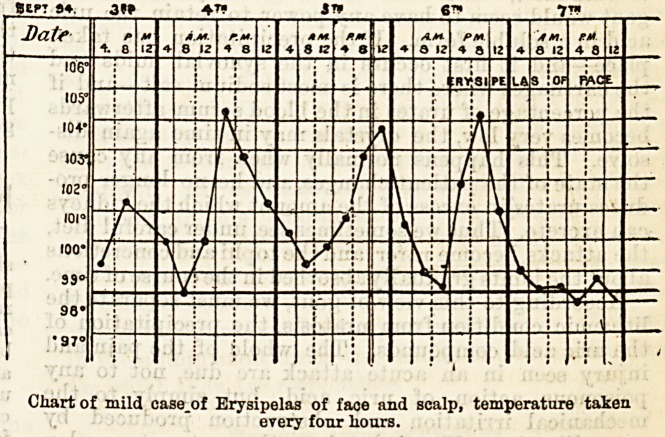# The Treatment of Erysipelas

**Published:** 1893-11-18

**Authors:** 


### ROYAL INFIRMARY, EDINBURGH.

### The General Treatment of Erysipelas.

Though somewhat vaguely defined, even in modern text-books, the term erysipelas may be taken to mean the specific contagious disease formerly described as the *cutaneous* form, the *phlegmonous* variety now more usually known as cellulitis, being probably an essentially different condition.

It has been regarded by some authorities as a general disease, with local symptoms; by others as a local disease with general symptoms, and for both sides there is much to be said; but whichever view is adopted there can be no doubt that the general condition of the patient is one requiring careful management, on which much of the success or failure of treatment depends.

After a variable incubation period, during which the patient suffers from malaise and nausea, frequently going on to actual vomiting, the temperature runs up to 103 deg. F., or even higher, and locally the well-known area of redness appears on the skin, frequently, when on the face, commencing at the angle of the eye or ala of the nose, while on the body its site is usually determined by the presence of a wound.

The affected area is raised and tender to pressure, and, while spreading, has a sharply defined margin, from which processes may be felt projecting into the surrounding subcutaneous tissue, chiefly in the direction of the lymphatics.

The patient presents all the features of an acute disease. The temperature ranges to 103 deg. F., and upwards, the pulse is rapid and soft, while there is usually thirst, more or less complete anorexia, and considerable gastric disturbance, not unfrequently vomiting. There is usually constipation, while the urine is concentrated, and may contain albumen.

After a variable time the area of redness ceases to spread and rapidly fades; this is followed by desquamation of the cuticle, these local changes being accompanied by a sudden fall of temperature, often to subnormal, and the attack is over. On the other hand, while the area of redness is spreading, each marked increase is accompanied by a rise of temperature, with intervening remissions. Though erysipelas tends towards spontaneous recovery, and does not usually end fatally, the prognosis is grave in infancy and old age, and also when complicated by renal disease.

With regard to treatment, one cannot hesitate as to the advisability of isolation, more especially when occurring in a surgical ward. In Edinburgh the cases are removed to a special block of buildings separated from the main hospital; on recovery, if readmitted to the wards, they are submitted to a process of quarantine in a special ward. The patient must be confined to bed in a well warmed apartment and warmly clad. Abundant liquid nourishment is required, of which milk constitutes the greater part, but beef tea or even whipped egg may be allowed, depending on the degree of gastric disturbance. Stimulants will be required in by far the majority of cases. In the case of an adult a tablespoonful of brandy or whiskey may be given every four hours, and this frequently requires to be increased. The free action of the bowels must be secured by the administration of a saline such as sulphate of magnesia from time to time, and in the simpler cases probably nothing more will be necessary. Many drugs have been recommended for the treated of erysipelas by internal administration of which perhaps the chief are iron, quinine and aconite. In Edinburgh the first of these is chiefly used in the form of the tincture of the muriate of iron (Edin: Pharmacopœia), the preparation originally recommended by Bell for this purpose. This preparation differs from the official tincture of the perchloride in containing a greater proportion of free acid, and is both pleasanter to take and causes less gastric trouble. It may be given in doses of ♏xx. and upwards every four hours.

In spite of the great reputation of iron, it may at least be doubted if the marked specific effect attributed to it by its early advocates can be sustained, and in many cases it seriously aggravates the condition of the stomach. In such cases the administration of bismuth, rhubarb, and soda may be desirable, or in more severe cases hydrocyanic acid and other sedatives are required. Quinine and aconite have been chiefly recommended for their antipyretic properties, but the former frequently causes vomiting, and the end can be more effectually attained by the use of the ice-cap.

Should heart failure threaten, it will be necessary to give some cardiac tonic, such as digitalis, although it frequently causes vomiting. The frequency of delirum

Chart of mild case of Erysipelas of face and scalp, temperature taken every four hours.

often makes it necessary to procure sleep, which, when milder measures fail, may generally be safely done by an injection of morphia.

Locally, the number of applications recommended in erysipelas is almost infinite, and the objects in view are also very varied. The method of using iodine, recommended by Dr. Miles, and described in a recent number of The Hospital, is a distinct advance in limiting the spread of the disease, while, when there is much pain, an ointment of icthyol in lanoline 20 per cent., applied on lint, gives great relief. Other methods resolve themselves chiefly into modes of protection, which is secured in a cleanly and efficient manner by a light wool dressing where it can conveniently be applied.

The point to keep in view is that the disease is a most exhausting one, and is of somewhat variable duration, so that any measure which will sustain the patient's powers is of value, otherwise the final fall of the temperature, which marks the end of the disease, may be the period when the real difficulties of treatment begin, and the patient, bankrupt in energy, threatens to die from asthenia. In more favourable cases after the crisis appetite returns, and in addition to liquid, a little fish and pudding, and after a day or so chicken may be allowed, while stimulants are reduced, and spirits partially or entirely replaced by wine and tonics. Throughout the whole course of the disease careful nursing and attention is of the utmost importance.

## Figures and Tables

**Figure f1:**